# Production of Toxic Volatile Trimethylbismuth by the Intestinal Microbiota of Mice

**DOI:** 10.1155/2011/491039

**Published:** 2011-10-10

**Authors:** Britta Huber, Philip Dammann, Christine Krüger, Petra Kirsch, Beatrix Bialek, Roland A. Diaz-Bone, Reinhard Hensel

**Affiliations:** ^1^Department of Microbioloy I, University of Duisburg-Essen, UniversitaetsstraBe 2, 45141 Essen, Germany; ^2^Central Animal Laboratory, University Hospital Essen, HufelandstraBe 55, 45122 Essen, Germany; ^3^Animal Research Center, University of Ulm, Oberberghof, 89081 Ulm, Germany; ^4^Instrumental Analytical Chemistry, University of Duisburg-Essen, UniversitaetsstraBe 2, 45141 Essen, Germany

## Abstract

The biotransformation of metals and metalloids into their volatile methylated derivatives by microbes growing under anaerobic conditions (e.g., the mammalian intestinal microbiota) plays an important role in spreading these compounds in the environment. In this paper, we could show that the presence of an intact intestinal microbiota of mice provides the *conditio sine qua non* for the production of these mostly toxic derivatives. To document the indispensible role of the intestinal microbiota in methylating metals and metalloids to volatile derivatives under *in vivo* conditions, we compared the methylation capability of conventionally raised (CONV) and germ-free (GF) B6-mice fed with chow containing colloidal bismuth subcitrate (CBS) as the starting material for the formation of volatile methylated metal(loid)s. Permethylated volatile trimethylbismuth ((CH_3_)_3_Bi) was only detected in the blood of the conventionally raised mice. Concomitantly, a higher bismuth concentration was found in organs such as liver, lung, testicles, and brain of the CONV mice as compared to those of GF mice (*P* > 0.01), strongly suggesting a correlation between the intestinal biomethylation of bismuth and its accumulation in mammalian tissues.

## 1. Introduction

The biotransformation of metals and metalloids into their volatile methylated derivatives raises ecological and health concerns, since this process increases the toxicity and distribution of the respective elements in our environment (e.g., As, Sb, Te, and Bi) [1–7]. Microorganisms, in particular methanoarchaea growing under anaerobic conditions (e.g., in wetlands, lake sediments, sewage sludge, landfills), are responsible for this process [2, 8–12]. Recent reports of our lab indicate that these transformations are also catalyzed by microbiota originating from the mammalian intestine: we detected the *ex situ* production of volatile metal(loid)s in human feces samples and in contents of isolated gut fragments of conventionally raised (but not of germ-free) mice after ingestion of bismuth containing pharmaceuticals [11] or in pure cultures of common gut methanoarchaea and bacteria [13]. As the toxicity of trimethylbismuth (TMBi) was found to be approximately two orders of magnitude higher in comparison to inorganic bismuth both in animal experiments [5] as well as in more recent studies on cytotoxicity [4], the production of methylated bismuth species by intestinal biomethylation is of potential health concern.

However, all studies to date concerning the role of the intestinal microbiota in the methylation of metals and metalloids were performed under *ex situ* conditions, physiological conditions within the living organisms have been disregarded so far. Our present study addresses this deficit by analyzing the role of the intestinal microbiota in transforming metal(loid)s to volatile methylated derivatives *in vivo* by comparative studies of conventionally raised (CONV) and germ-free (GF) mice fed with colloidal bismuth subcitrate (CBS) containing chow. After a feeding period of 14 days, the formation of volatile bismuth compounds was followed by determining their content in the blood of the animals using purge-and-trap gas chromatography hyphenated to element-specific detection by inductively coupled plasma mass spectrometry (PT-GC/ICP-MS). Moreover, the effect of the intestinal microbiocenosis on the uptake of bismuth was studied by analyzing the total content of bismuth in the organs. Conventionally raised mice, which were not fed with bismuth containing chow, were used as control group.

Bismuth was used as model starting material for metal(loid) methylation in the present study because of its common presence in our environment due to its importance in technological application (e.g., as additive in cosmetics, catalysts, industrial pigments, alloys and ceramics [14]) and in health care (as antimicrobial agent in pharmaceuticals for the treatment of peptic ulcers caused by *H. pylori*). For a long time, bismuth was thought to be a less toxic element but since the 1970s it is well accepted that high-dosed medical treatment with bismuth subgallate and subnitrate can cause severe encephalopathies, renal failure, and genotoxic effects [15–17]. However, the clinical use of CBS does not appear to cause neurotoxicity [18].

## 2. Materials and Methods

### 2.1. Handling and Feeding of the Mice

Feeding experiments were conducted with CONV and GF B6-mice (Mus musculus, males, ages 12 to 17 weeks). All experiments were carried out in accordance with the German animal welfare act for animal experiments. Animal husbandry for CONV mice was performed by the Central Animal Laboratory of the University Hospital Essen, and GF mice were bred in the Animal Center of the University of Ulm and kept under sterile conditions in a germ-free isolator.

The animals were housed individually in standard Macrolone cages type III (38 by 22 by 15 cm) with Softwood bedding material (type S 3/4, Sniff GmbH, Soest, Germany) under standard laboratory conditions (12-h light-12-h dark cycle with lights on at 8 a.m.; temperature, 21 +/− 1°C; relative humidity, 55% +/− 10%). Both CONV and GF mice were fed with an autoclaved, standard diet enriched with colloidal bismuth subcitrate (CBS) obtained from De-Noltab (Yamanouchi Europe B.V., The Netherlands) with a final bismuth content of 50 mg kg^−1^ (SNIFF V1534Bi, SNIFF GmbH, Soest, Germany). The diet and bottled tap water were available *ad libitum*. The bismuth uptake of mice during the bismuth-enriched diet was calculated to be approximately 8.3 mg kg^1^ day^−1^. Conventionally raised mice fed with a autoclaved, commercial standard mouse diet without CBS (SNIFF V1534) served as control animals.

The feeding experiments were conducted for 14 days in three groups:

- CONV control mice (three individuals) fed with standard diet,
- CONV mice divided in 8 subgroups with 3 individuals each (24 CONV mice fed with a bismuth-enriched diet),
- GF mice divided in 7 subgroups with 3–5 individuals each (28 GF mice fed with bismuth-containing chow).

### 2.2. Sampling of Blood and Organ Tissues of the Mice

After 14 days of feeding with standard or CBS-containing diet, the mice were anesthetized with a ketamine/xylazine-narcosis (120 mg kg^−1^ body weight or 13 mg kg^−1^ body weight, resp.; i.p.). The blood was extracted via cardiac puncture under sterile conditions and transferred immediately into autoclaved, helium-filled, and gas-tight-sealed 120 mL glass vials containing A. bidest with 1‰  anti-foaming agent (antifoam 289 Sigma-Aldrich, Taufkirchen, Germany) and heparin (750 IE mL^−1^, Ratiopharm, Ulm, Germany). To increase the sample volume for a reliable determination of the volatile derivative, the blood of three to five mice was pooled in subgroups with a total volume of 3-4 mL blood. After cervical dislocation, the mice were dissected and the organs (liver, lung, kidneys, testicles, and brain) were removed and stored at −80°C until analysis of total bismuth concentration in these tissues. Speciation analysis was performed within 12 h after sampling.

### 2.3. Speciation and Quantification of Volatile Methylated Bismuth

The volatile bismuth in the headspace of the blood samples was analyzed using a modified purge-and-trap gas chromatographic system (PT-GC) coupled to an inductively coupled plasma mass spectrometer (ICP-MS) (Fisons VG, Plasma Quad II) as an element-specific detector as described previously [8]. The bismuth species eluting from the GC column were detected online by ICP-MS at a mass/charge ratio of *m/z* 209, and the identity of the detected volatile species was ensured by boiling point chromatographic retention time correlation (boiling point of (CH_3_)_3_Bi at 760 mmHg = 108.8°C resulting in a retention time of 150 s under the given conditions). The limit of detection (LOD) of the analyte was calculated with the 3*σ* criterion based on the noise of the baseline, that is 1.5 fmol for bismuth. The whole headspace of the vials (~105 mL depending on the volume of the extracted blood) was purged and trapped for five minutes. The completeness of the (CH_3_)_3_Bi removal from the blood was verified by repetition of the purge and trap procedure. The content of (CH_3_)_3_Bi was calculated per g blood wet weight.

### 2.4. Total Metal Analysis of Organ Tissues

The bismuth content of the organs was determined after homogenizing the tissues using the Microdismembrator S (Sartorius, Göttingen). The homogenates were dried at 110°C to constant weight. Aliquots (0.1 to 0.5 g) of the dried homogenates were suspended in 4 mL HNO_3_ (65%, subboiled) and 2 mL H_2_O_2_ (30%, Suprapur; Merck, Darmstadt, Germany), digested in a microwave-accelerated reaction system (MARS 5; CEM, Kamp Lintfort, Germany) and analyzed by ICP-MS-analysis as described previously [11]. Total metal content was referred to g dry weight. For statistics, the nonparametric Mann-Whitney-Wilcoxon *U* test was used.

## 3. Results and Discussion

### 3.1. The Transformation of Bismuth to Its Permethylated Volatile Derivative (CH_3_)_3_Bi in Mice Requires an Intact Intestinal Microbiota

To evaluate the role of the intestinal microbiota of mice in transforming metal(loid)s into their volatile methylated derivatives under *in vivo* conditions, we determined the content of volatile bismuth compounds in the blood of CONV mice (i.e., mice with intact intestinal microbiota) compared to that of GF mice, both fed with CBS-enriched chow. For that purpose, the blood of the mice was extracted via cardiac puncture and analyzed for volatile bismuth compounds after a feeding period of two weeks. As shown in Table 1, permethylated (CH_3_)_3_Bi was the only volatile compound and it could exclusively be found in the blood of CONV fed with CBS-enriched chow mice. Neither in the blood of GF mice fed with CBS-enriched chow nor in the blood of CONV mice fed without CBS-enriched chow (CONV control mice) (CH_3_)_3_Bi could be observed. This result clearly indicates that the presence of the intestinal microbiota is indispensible for the transformation of metal(loid)s into their volatile-methylated derivatives. Most probably, the microbiota itself is responsible for the transformation under *in vivo* conditions as already suggested by various *ex situ* analyses of cultures derived from the intestinal microbiota [11, 13]. We cannot, however, exclude that epithelial and/or mesenchymal cells of the mice partly contribute to these transformation processes under *in vivo* conditions implying, however, that the intact intestinal microbiota induces such an activity. 

Previous studies of intestinal biovolatilization of bismuth in human fecal samples and in human breath after ingestion of bismuth have shown an extremely high interindividual variability of 5 [11] and, 3 orders of magnitude [19], respectively. In this study, the variation of the (CH_3_)_3_Bi content observed in the blood of the different subgroups of the CONV mice is in accordance with previous studies [11] ranging from 6.2 to 71.4 fmol g^−1^ w.w. with a mean value of 21.6 ± 22.1 fmol g^−1^ w.w. (*n* = 8). Considering the highly standardized conditions of animal husbandry applied in this study, this relatively high variability probably reflects differences in the composition of the intestinal microbiota and its metabolic activity across the test population.

### 3.2. Methylation of Bismuth Is Accompanied with an Accumulation of Bismuth in Organ Tissues

**Figure 1:**
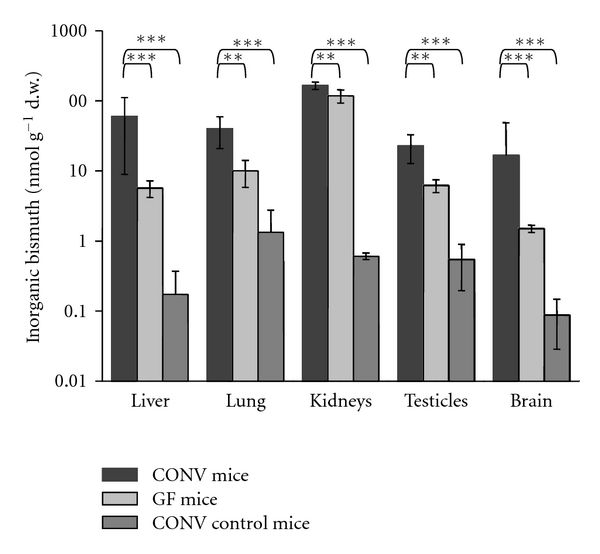
Bismuth content (means ± SD (nmol g^−1^ d. w.)) of organ tissues of CONV mice and GF mice fed with CBS containing chow as well as that of the CONV control mice fed with a standard diet. (Significance levels: ***P < 0.001, **P < 0.01.)

To examine the impact of the methylation on the absorption of bismuth by the mammalian organism, which could cause toxic effects of the bismuth derivatization, the bismuth content of liver, lung, kidneys, testicles, and brain of CONV and GF mice was investigated by microwave digestion and subsequent analysis by ICP-MS. Interestingly, the bismuth content of all organ tissues tested was significantly higher in CONV mice as compared to GF mice (Figure 1), indicating that the intestinal microbiocenosis increases the bioavailability of bismuth from the intestine. While mobilization of bismuth by chelation would presumably play a major role in this process, the formation of methylated bismuth species produced in the CONV mice can significantly contribute. At first, methylated metal(loid) species can more easily permeate through cell membranes due to their higher hydrophobicity—accumulated in the cell by internal complexation with various compounds—and thus permanently detracted from the blood. Next, the formation of permethylated trimethylbismuth proceeds via partly methylated mono- and dimethyl bismuth, which are not accessible via GC-ICP-MS due to their low volatility, but can also contribute to the increased bioaccessibility of bismuth due to biomethylation.

The comparably high content of bismuth in the kidneys of both CONV mice and GF mice is probably due to the fact that the main part of inorganic bismuth is excreted via the urinary system in mammals [17]. In the organ tissues of the CONV control mice fed with chow without bismuth addition, only low amounts of bismuth close to the LOD were detected as expected for ICP-MS-analysis.

## 4. Conclusion

The observation that only CONV mice possessing an intact intestinal microbiota but not GF mice are able to transform bismuth to its permethylated derivative (CH_3_)_3_Bi proves the indispensable role of the intestinal microbiota in methylating metals and metalloids to their volatile derivatives in the living organism.

The detection of volatile (CH_3_)_3_Bi in blood of CONV mice fed with CBS containing chow could be of some relevance for healthiness. Obviously, the methylation of bismuth to its volatile derivative (CH_3_)_3_Bi promotes the dispersal of the metal in the mammalian organism via the blood resulting in a significant accumulation of bismuth in organ tissues, presumably due to its increased hydrophobicity caused by methylation, which increases interactions with and penetration of cell membranes. To evaluate the resulting health risk, the bismuth species accumulated in tissues throughout the organism and its inter- and intracellular interactions has to be determined.

## Figures and Tables

**Table 1:** (CH_3_)_3_Bi content in blood samples of CONV and GF mice fed with CBS containing chow and CONV control mice on standard diet. The absolute limit of detection (LOD) for (CH_3_)_3_Bi based on the 3*σ* criterion was 1.5 fmol, whereas the relative LOD was between 4.5 and 6 fmol g^−1^.

CONV mice		GF mice		CONV control mice	
Subgroup	fmol g^−1^ (w. w.)	Subgroup	fmol g^−1^ (w. w.)	Subgroup	fmol g^−1^ (w. w.)
1	15.1	1	LOD	1	LOD
2	16.8	2	LOD	2	LOD
3	71.4	3	LOD	3	LOD
4	8.4	4	LOD		
5	6.2	5	LOD		
6	35.1	6	LOD		
7	12.3	7	LOD		
8	7.4				

## Abbreviations

LOD: Limit of detection
(CH_3_)_3_Bi: Trimethylbismuth
CBS: Colloidal bismuth subcitrate
CONV mice: Conventionally raised mice
GF mice: Germ-free mice
PT-GC system: Purge-and-trap gas chromatographic system
ICP-MS: Inductively coupled plasma mass spectrometer.
